# *Giardia *assemblage A: human genotype in muskoxen in the Canadian Arctic

**DOI:** 10.1186/1756-3305-1-32

**Published:** 2008-09-22

**Authors:** Susan J Kutz, RC Andrew Thompson, Lydden Polley, Kami Kandola, John Nagy, Caroline M Wielinga, Brett T Elkin

**Affiliations:** 1 Department of Ecosystem and Public Health, Faculty of Veterinary Medicine, University of Calgary, Calgary, Alberta, Canada; 2 World Health Organization Collaborating Centre for the Molecular Epidemiology of Parasitic Infections, School of Veterinary and Biomedical Sciences, Murdoch University, Murdoch, WA, 6150, Australia; 3 Department of Veterinary Microbiology, Western College of Veterinary Medicine, University of Saskatchewan, Saskatoon, Saskatchewan, Canada; 4 Health Protection and Promotion Office, Stanton Territorial Health Authority. P.O. Box 10, Yellowknife, NT, Canada; 5 Environment and Natural Resources, Government of the Northwest Territories, NT, Canada

## Abstract

As part of an ongoing program assessing the biodiversity and impacts of parasites in Arctic ungulates we examined 72 fecal samples from muskoxen on Banks Island, Northwest Territories, Canada for *Giardia *and *Cryptosporidium*. *Cryptosporidium *spp. were not detected, but 21% of the samples were positive for *Giardia*. Sequencing of four isolates of *Giardia *demonstrated *G. duodenalis*, Assemblage A, a zoonotic genotype.

## Findings

'Spill-over' of pathogens from people and/or domestic animals to wildlife is increasingly recognized as a significant source of disease in wildlife populations [1]. Interspecies transmission and disease emergence are not unexpected in disturbed landscapes with high densities of people and domestic animals encroaching on wildlife habitat, but in remote and relatively pristine environments these events are unexpected and unexplored. Here we report the discovery of *Giardia duodenalis *Assemblage A in muskoxen (*Ovibos moschatus*) in a remote region of the Canadian Arctic and discuss the possible flow of this parasite in this ecosystem.

Banks Island, approximately 70,000 km^2^, is the western-most island of the Canadian Arctic archipelago. The permanent human population of approximately 120 is restricted to the community of Sachs Harbour on the southwest coast. There is limited tourism with fewer than 100 visitors to the island annually. Mammalian biodiversity is low, comprising approximately 50,000 muskoxen, 1100 Peary caribou, numerous arctic hares, arctic fox and brown and collared lemmings, polar bears, and the occasional grizzly bear (J. Nagy, unpubl. data).

72 fresh muskox fecal samples collected from the ground near Sachs Harbour during early August, 2004 were refrigerated until analysis for *Giardia *and *Cryptosporidium*. The method used by McAllister et al. [2] was modified as follows: 2 g of feces were suspended in 8 ml PBS; the suspension was strained through 2 layers of cheesecloth; centrifugation was at 300 × g; for each sample a 15 μl aliquot from each 1 ml of re-suspended deposit was placed in each of the two wells on an Esco Fluorescent microscope slide (Esco Products, Oakridge, New Jersey); Giardi-a-Glo fluorescent antibody was added to one well, and Crypt-a-Glo antibody to the other (Waterborne Inc, New Orleans, Louisiana); slides were incubated for 45 minutes at 37C. All fluorescing *Giardia *cysts and *Cryptosporidium *oocysts were counted.

*Giardia *was found in 15 of 72 (21%) samples. The median cyst count was 6,933/gram feces wet weight (range 133–348,533), or approximately 7 million cysts/muskox/day. All samples were negative for *Cryptosporidium *spp.

DNA was extracted directly from five positive samples maintained in ethanol using a QIAamp stool extraction kit (Qiagen, Doncaster, Australia) with modifications [3]. A nested PCR was used for amplification at the *18SrDNA *locus. For the primary reaction, primers RH11 and RH4 [4]were used and for the secondary reaction primers GiarF and GiarR [5]were used. Amplification conditions for primers varied slightly from published methods. For the primary PCR the reaction volume was increased to 37.5 μL or 50 μL, primer concentration was increased to 750 nM, magnesium concentration decreased to 1.5 mM, bovine serum albumin (BSA) was added to a final concentration of 0.5% and cycling conditions included a touch down. The reaction mixture contained 1.5 – 2.0 μL of extracted DNA, 1 × reaction buffer (67 mM Tris-HCl, 16.6 mM (NH4)2SO4, 0.45% Triton X-100, 0.2 mg/mL gelatin), 1.5 mM MgCl2, 750 nM of each primer, 200 μM of each dNTP, 1.0 unit of Tth+ DNA polymerase (Biotech International, Perth, Australia), 0.5% bovine serum albumin (BSA) and 5% dimethylsulfoxide (DMSO) in H2O.

Reactions were kept on ice until the thermocycler reached temperature and then denatured at 96C for 5 min followed by a touch down of 10 cycles (-0.5C/cycle) of 96C/30 sec, 58C-53C/45 sec, 72C/45 sec and a further 40 cycles 96C/30 sec, 53C/45 sec, 72C/45 sec, 1 cycle of 72C/7 min and 15C hold using a GeneAmp PCR System 2400 thermocycler (Perkin-Elmer, Waltham, U.S.A). The secondary PCR reaction conditions were similar to the primary, without the inclusion of BSA and DMSO. 1.5 μL of primary product was transferred into the secondary reaction mixture (37.5 μL final volume) and kept on ice until denaturation at 96C for 5 min followed by 40 cycles of 96C/30 sec, 53C/20 sec, 72C/30 sec, 1 cycle of 72C/7 min and a 15C hold. Products were isolated from agarose gel using a DNA purification kit (Mo Bio, UltraClean GelSpin, Carlsbad USA) as per manufacturer's instructions, except for a reduced elution volume.

Sequencing reactions were performed without DMSO, using Big Dye Terminator v3.1 cycle sequencing kit (Applied Biosystems, Scoresby, Australia) as per manufacturer's instructions. PCR products were sequenced in both directions. Reactions were electrophoresed on an ABI 3730 48 capillary machine. Sequencing profiles were analysed using SeqEd v1.0.3 (Applied Biosystems, Scoresby, Australia). Isolates were grouped into genetic Assemblages based on polymorphisms within the 130 base pair sequence [4,6]).

Cysts from four of five samples were identified as *Giardia duodenalis *Assemblage A and all sequences aligned to the Portland 1 reference isolate (Accession number M54878) with 100% homology over the region examined. Cysts from the fifth sample were not genotyped because of inhibition. Although more detailed analysis at a secondary locus was needed to identify the subgroup pattern, no further molecular characterization was possible on the remaining samples because of the amount of inhibiting compounds. The PCR additive BSA, as well as a range of dilutions and volumes, were used to moderate the effect of these compounds, however, ultimately insufficient sample was left for further analyses.

Discovery of *G. duodenalis *Assemblage A in muskoxen in this remote, frigid and sparsely populated region was unexpected. Published records of Assemblage A in free-ranging ungulates are limited to a white-tailed deer in Maryland[7], a roe deer from the Netherlands[8]and moose and reindeer in Norway [9]). Assemblage A in animals is typically associated with ongoing or historical contact with people (e.g., apes[10]; beavers[11]; dogs[12]), and we suspect that Assemblage A in muskoxen also resulted from a host-switch from people. This could have occurred once or repeatedly any time since the first known human presence on Banks Island (Pre-Dorset culture – 1500BC-1000AD). Muskoxen were extremely rare on the island in the early 20^th ^century, however, and the present infection may more likely result from recent, and perhaps ongoing, introduction(s) of *Giardia *associated with contemporary settlement and/or tourism. Previous studies elsewhere have demonstrated that people and their pets entering wilderness areas are often infected with *Giardia*[13]. Notably, the parasite has been reported, but not typed, in people in the NWT (255 cases from 1989–2006) (Kandola, unpubl. data) and in the eastern Arctic (40% prevalence in ages 0–4 and 17% overall)[14].

The high prevalence and intensity of *Giardia *observed in muskoxen in this study are unprecedented among wild ungulates and suggest that muskoxen are important hosts on Banks Island. Several characteristics of muskoxen and *Giardia *may support parasite maintenance. Muskoxen are highly susceptible and competent hosts for pathogens from many species[15]. They are relatively sedentary herd animals that congregate in river valleys for feeding, a behavioural trait contributing to large numbers of cysts in a moist environment and ongoing parasite exposure. Finally, *Giardia *cysts are cold tolerant, immediately infective, and few are needed to establish infection. Thus, muskoxen may now maintain *Giardia *Assemblage A in the absence of reservoir hosts or ongoing introductions and may serve as a source for infection for other species. Untyped *Giardia *sp. previously reported in Peary caribou on Banks Island (3% prevalence, Nagy, Larter, Olson unpubl. data), may represent spill-over from muskoxen.

Behaviour of people and muskoxen also affords several opportunities for ongoing transmission between these hosts and between terrestrial and marine systems. In summer, spatial overlap between residents of Sachs Harbour, tourists, and muskoxen is common, as all tend to concentrate around major water bodies. High abundance of *Giardia *infection in the large muskox population results in significant contamination of water bodies and, because latrines are rare in this vast landscape, contamination of the land and water-bodies with human feces is likely. Residents and tourists often drink untreated water directly from these water bodies. Human sewage from Sach's Harbour, deposited untreated in a sewage pond accessible to wildlife, provides ongoing opportunities for parasite dispersal, as do the commercial muskox harvests that are held almost every winter near the community. People working at these harvests may be directly exposed to cysts in the offal. Additionally, for many years this offal was disposed of untreated on the land near the temporary abattoirs used for the harvests, and more recently has been deposited on the sea ice. These disposal practices may provide a source of infection for people and terrestrial and marine wildlife. Assemblage A has been detected in marine mammals in the western Arctic [16](Olson, unpubl. data), but the significance of offal disposal on sea ice is unknown.

*Giardia *is an important pathogen in people and domestic animals, however, the literature on effects in wildlife remains sparse. In the harsh and rapidly changing arctic environment, the impacts of *Giardia*, together with cumulative effects of other pathogens, weather events, and directional climate change, on the health and sustainability of muskox populations deserves further consideration.

Molecular parasitology allowed us to rapidly identify a potential zoonotic pathogen circulating among muskoxen in a remote region of the Arctic. Further samples and analyses are required from both the muskoxen and the local people to determine sub-types and transmission patterns among these hosts. Nevertheless, our findings, together with similar findings of Assemblage A in reindeer and moose in Norway [9], leave many questions regarding the origin of *Giardia *in arctic/subarctic wildlife, its health impacts, and the patterns of parasite flow among people and wildlife and among terrestrial and marine systems. Much, but not all (see [13])) of the literature on *Giardia *in wildlife is limited to surveys that, typically through correlation, implicate wildlife as a source of infection in people. Our results emphasize the need to adopt a less anthropocentric view of *Giardia *and to re-explore the complexities of interspecies transmission and the behavioural, cultural, and environmental factors that influence the introduction and flow of *Giardia *among people and wildlife. The relatively simple arctic ecosystems provide an opportunity to gain significant insights into disease transmission among people and wildlife and thereby improve our understanding of infectious disease in more complex systems.

## Competing interests

The authors declare that they have no competing interests.

## Authors' contributions

SK, JN, BE, and LP conceived of the study. AT and CW carried out the molecular genetic studies. KK contributed data and expertise in human aspects of *Giardia*. SK and LP drafted the manuscript, all authors contributed significantly to editing the manuscript. All authors read and approved the final manuscript.
